# Limited genome evolution of *Cryptococcus neoformans* following an accidental infection in the research laboratory

**DOI:** 10.1128/asmcr.00209-25

**Published:** 2026-02-03

**Authors:** Yunfan Fan, Madhura Kulkarni, Sean X. Zhang, Winston Timp, David J. Sullivan, Daniel F. Q. Smith, Arturo Casadevall, J. Marie Hardwick

**Affiliations:** 1 Biomedical Engineering Department, Johns Hopkins University 1466, Baltimore, Maryland, USA; 2 W. Harry Feinstone Department of Molecular Microbiology and Immunology, Johns Hopkins Bloomberg School of Public Health 25802 , Baltimore, Maryland, USA; 3 Division of Medical Microbiology, Department of Pathology, Johns Hopkins University School of Medicine 1500 , Baltimore, Maryland, USA; 4 Microbiology Laboratory, Johns Hopkins Hospital 588543, Baltimore, Maryland, USA; 5 Department of Pharmacology and Molecular Sciences, Johns Hopkins University School of Medicine 1500 , Baltimore, Maryland, USA; 6 Department of Neurology, Johns Hopkins University School of Medicine 1500, Baltimore, Maryland, USA; 7 The Sidney Kimmel Comprehensive Cancer Center, Johns Hopkins School of Medicine 1500, Baltimore, Maryland, USA; Rush University Medical Center, Chicago, Illinois, USA

**Keywords:** accidental inoculation, primary cutaneous cryptococcosis, genome evolution, needle stick, *Cryptococcus*, *neoformans*

## Abstract

**Background:**

Cutaneous infections resulting from accidental exposure to fungal pathogen *Cryptococcus neoformans* in research laboratories are rarely reported in the literature. To fill a gap in published guidance for handling these situations promptly when they arise, we describe a case report plus three additional examples as a guide to effective resolution.

**Case Summary:**

An immunocompetent laboratory researcher developed primary cutaneous cryptococcosis following accidental infection with *Cryptococcus neoformans* H99 via a needle scrape that was initially assumed not to have penetrated the skin. On day 8 after the incident, a characteristic nodular erythematous lesion developed on a finger at the exposure site. Antibacterial therapy was initiated for the clinical impression of bacterial cellulitis as clinical tests for cryptococcal serum antigen and cultures of aspirates from the wound site on day 13 after incident were negative. Eight-week fluconazole therapy initiated on day 15 after the incident was curative. Whole genome sequencing of yeast grown from wound exudate collected on day 18 confirmed high sequence similarity to H99 *Cryptococcus neoformans* var. *grubii* and sequence divergence from an archived sample isolated from an immunocompromised individual. Three additional cases with potential exposures to *C. neoformans* in the research lab received prophylactic fluconazole and remained asymptomatic.

**Conclusion:**

In a span of 5 years, four laboratory researchers from two research groups at the same institution sustained accidental exposures to *C. neoformans*, and all were successfully treated. These cases reinforce and update the recommendations for initiating antifungal therapy immediately after laboratory accidents involving *C. neoformans*.

## INTRODUCTION

*Cryptococcus neoformans* is a common environmental fungus, an opportunistic pathogen, and a significant public health burden (1). *C. neoformans* infections are assumed to be acquired by inhalation of fungal spores and can cause severe pulmonary and central nervous system disease in immunocompromised hosts (2). In addition to brain infections, the skin is another common manifestation of disseminated cryptococcosis (secondary cutaneous infection) (3). In contrast, primary cutaneous cryptococcal infections of immunocompetent and immunocompromised individuals are rare, and there are relatively few well-documented cases in the literature (4–6). Immunocompetent individuals typically acquire *Cryptococcus* sp. from their environment through skin abrasions during outdoor work (7). An underrecognized risk of accidental skin exposure occurs during laboratory research on *Cryptococcus*. Although accidental inoculation of a wide range of infectious agents via contaminated needles or other implements is a known hazard for health-care workers, we found only two well-documented reports of accidental inoculation with *Cryptococcus* in clinical settings (8, 9) and only a single report over 30 years ago of cutaneous cryptococcosis acquired in the research laboratory (10). In that case, a symptomatic lab worker was successfully treated with flucytosine (2 g/day for 4 weeks). In the current case, firsthand recognition that minor exposures to *C. neoformans* can result in cutaneous infections that require intervention was the motivation for this update on management of occasional accidental exposures or potential exposures in the research laboratory. This documented case of human-passaged *C. neoformans* was further analyzed for genome sequence evolution compared to the reference genome (H99) and a clinical isolate of *C. neoformans* from an AIDS-associated case.

## CASE PRESENTATION

### Case 1

**Fig 1:**
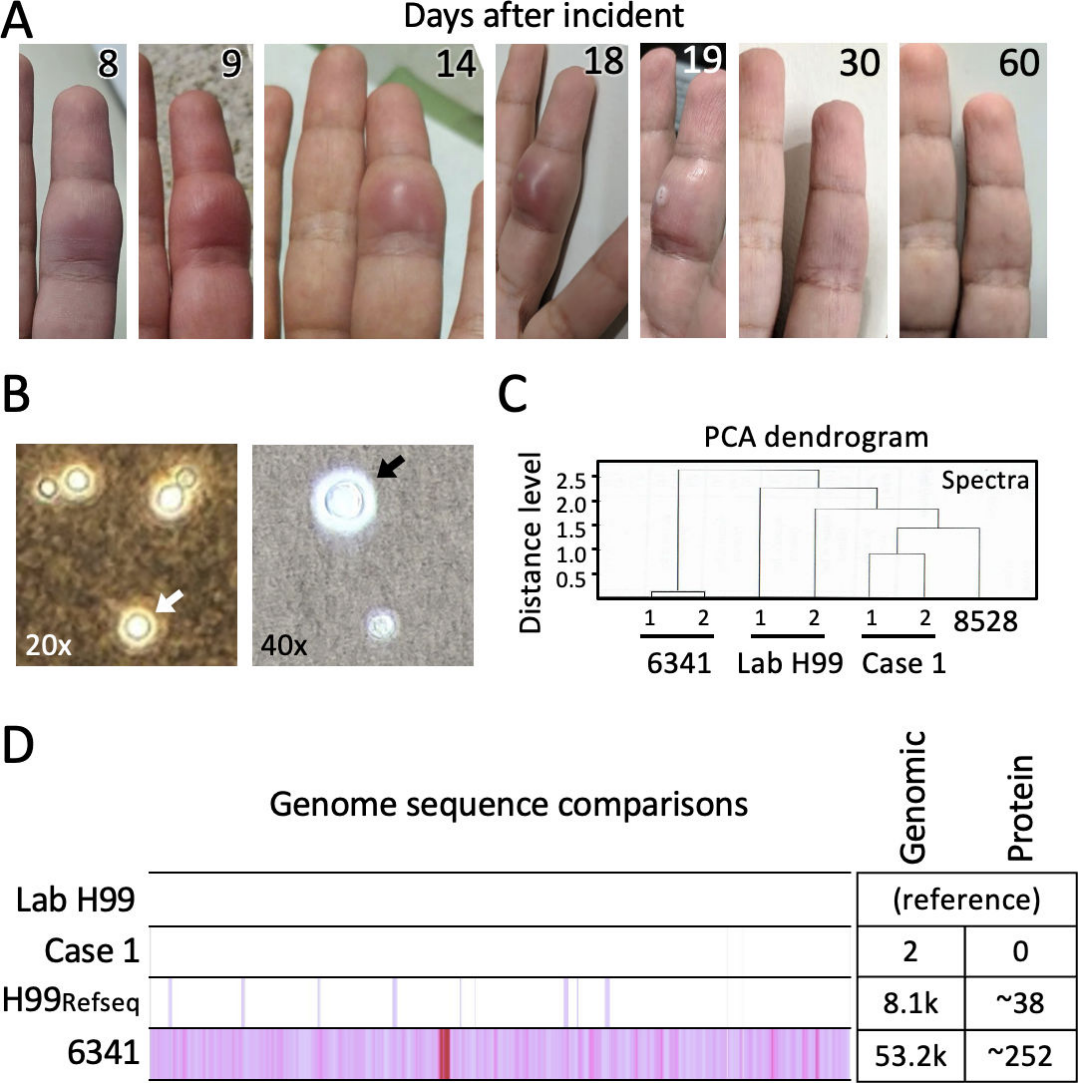
Cutaneous cryptococcosis in a laboratory researcher following exposure to a contaminated needle (case 1). (**A**) Presentation of primary cutaneous cryptococcosis on days 8, 9, 14, and 18 before draining, day 19 after spontaneous draining, 1 and 2 months after the incident. A pair of pinholes where clinical aspirates were taken is visible in day 14–19 images. (**B**) India ink staining of yeast-like colonies grown from exudate recovered from spontaneous wound drainage on day 18 revealed budding and non-budding yeast present in culture surrounded by unstained capsule (arrows) indicative of *Cryptococcus* sp. (**C**) Proteomic spectrum MALDI-TOF MS typing results comparing two clinical patient isolates of *Cryptococcus neoformans* var. *grubii* from immunocompromised patients at the Johns Hopkins Hospital Mycology Archive (6341 and 8528), lab strain H99, and case 1 (tested in duplicate, except 8528), indicative of low divergence between all samples. (**D**) Whole genome Illumina sequencing revealed that case 1 was highly related to lab strain H99 from which it was derived and more distantly related to a clinical patient isolate (6341). Reads were aligned to the *C. neoformans* var. *grubii H99* reference genome (GCA_000149245.3) using Bowtie2. Variants were called using FreeBayes (reported here for the coding mRNA strand), and variant effects were determined using SnpEff. Custom scripts were used to filter and count variants with at least 15× read coverage and ≥50% of reads supporting the non-reference allele. Differences relative to the lab strain of H99 understudy in case 1 are shown. The diagram approximates the genomic differences between indicated strains.

A laboratory researcher (case 1) presented with a lesion on the left ring finger 10 days after suspected laboratory exposure to the H99 laboratory strain of *C. neoformans*. The laboratory incident involved a needle abrasion that occurred while infecting insect larvae (*Galleria mellonella*), a commonly used non-vertebrate animal model for pathogenesis research (11). Although the exposure incident was marked by a sharp sensation, immediate inspection detected no bleeding or obvious signs of a breach to the skin or to the double gloves, and therefore no treatment was initiated. Redness appeared at the site on day 8 after the incident, and swelling increased on day 9 post-exposure (Fig. 1A). On day 13, the lab worker reported left arm axillary lymph node pain on days 10–12. The lab worker was not febrile and did not complain of headache or exhibit evidence of disseminated infection or systemic illness, and the nodular erythematous infection site was not painful initially. Clinical aspirates of a few microliters taken from two adjacent points on the swollen site were negative for bacterial and fungal growth, although the samples were not evaluated microscopically. Serum cryptococcal antigen was negative, and complete blood counts were normal. The lab worker was prescribed a 2-week course (days 13–29 post-incident) of sulfamethoxazole and trimethoprim by occupational health services for the clinical impression of bacterial cellulitis but without improvement. After further clinical consultation with infectious disease physicians, the lab worker started a 4-week course of 200 mg/day fluconazole (days 15–42).

One day after initiation of antifungal therapy (day 16), the lesion formed an abscess at the sites where clinical aspirates had been taken and became painful. The abscess drained spontaneously 2 days later (day 18), and axial lymph node pain recurred (days 23–29 post-incident). At the 4-week follow-up exam, swelling was reduced, but discoloration persisted. The lab worker received an additional 4 weeks of 200 mg/day fluconazole (days 43–70) for a total of 8 weeks. Liver enzymes were all within normal limits. After 6 weeks of treatment, swelling and discoloration at the site subsided and returned to normal by 60 days post-incident (Fig. 1A). In the subsequent 5 years, the lab worker remained healthy with no evidence of persistent infection or other sequelae.

On day 18 while traveling, the lab worker collected several microliters of exudate from the draining abscess in an unused zip-top bag and stored the specimen in a mini-frig before transport to the research lab. The specimen was plated on yeast peptone dextrose (YPD) agar plates and incubated at 30°C for 2 days, which resulted in growth of yeast-like colonies. Yeast cells collected from YPD plates were India ink test positive, indicative of cryptococcal capsule halos (Fig. 1B). The growth was transferred to the clinical mycology lab, which identified the exudate-derived yeast as *C. neoformans* by MALDI-TOF MS (Fig. 1C) (12) and further identified this human-passaged isolate as *C. neoformans* var. *grubii* H99 alpha type A by whole genome sequencing performed in parallel with the H99 lab strain used by the lab worker (Fig. 1D). Of the ~3,000 initial SNPs called, only two nucleotide changes in non-coding regions of two uncharacterized genes were identified in filtered assemblies (<0.00001% change). Human-passaged *C. neoformans* (case 1) had an intronic A deletion within a string of adenines (A_12_ > A_11_, in 58 of 59 reads) adjacent to the junction of intron-4 and exon-5 of CNAG_02125. The second change was a non-coding G>C present in 52% of reads, along with other non-coding changes at lower frequencies in CNAG_05712, which are also present at similar frequencies in the archival clinical isolate *C. neoformans* 6341 from a patient with HIV-AIDS sequenced in parallel. No changes in the human-passaged isolate are predicted to alter splicing or protein sequence.

The closely related H99 lab strain and the case 1 isolate each differ by ~8,000 sequence changes (0.04%) from the official H99 reference genome (GCA_000149245.3) isolated over 45 years ago from a patient with Hodgkin’s lymphoma, reflecting a relatively low evolution rate in the laboratory. In contrast, both the human-passaged isolate (case 1) and the corresponding H99 lab strain are more distantly related to the HIV-AIDS-associated isolate (6341) that differed by ~53,000 nucleotides (0.28%) (Fig. 1D), consistent with similar divergence in other studies (13). The observed evolution of *C. neoformans* over 18 days of human-passaged H99 *C. neoformans* in case 1 is considered inconsequential.

### Cases 2–4

Within a few weeks of the incident for case 1, another researcher in a second research laboratory in the same department (case 2) received a penetration injury from broken glassware contaminated with *C. neoformans*. The researcher received prophylactic fluconazole (200 mg/day for 14 days) starting within 24 h of sustaining the injury and did not develop a skin infection. In the same lab as case 2, a third incident occurred approximately 3 years later (case 3). Like case 1, a lab researcher was exposed to *C. neoformans* by an accidental stick with a contaminated needle (while recapping) during experiments with *G. mellonella* larvae. No yeast could be recovered from the injection site. Case 3 received prophylactic fluconazole (200 mg/day for 30 days) beginning on the day of exposure and did not develop signs of infection or disease. A clinical blood sample taken immediately following exposure was negative for *Cryptococcus* via PCR, and liver enzymes were normal. While *G. mellonella* larvae are a useful model of invertebrate *C. neoformans* pathogenesis (11), they pose increased risk of accidental needle stick exposure to laboratory researchers during injection procedures.

Approximately 2 years later, a fourth laboratory researcher from the same lab as case 1 expressed concern of a potential exposure while working with *C. neoformans* during a mouse infection experiment (case 4). Within 24 h, the researcher was prescribed prophylactic fluconazole (200 mg/day for 2–4 weeks) and did not develop symptoms.

## DISCUSSION

Disseminated *C. neoformans* infections can manifest as secondary cutaneous cryptococcosis in individuals with immune-related disorders, organ transplant, immunosuppressants, some cancers, and 5%–15% of AIDS cases (14–18). In contrast, primary infections often involve outdoor occupations or exposure to avian excrement, although the inoculation event is often not determinable (4, 5, 7). Rare primary cryptococcal infections of immunocompetent individuals acquired through skin abrasions are challenging to diagnose (19, 20), as encountered in this case study of four laboratory researchers.

In case 1, the researcher was initially uncertain if an injury had occurred in the laboratory or outside of the lab, which included potential injuries while transporting boxes and furniture over the subsequent weekend. This uncertainty was confounded by difficulty in diagnosing cutaneous cryptococcosis lesions in the clinic using standard tests. Despite the prevalence of accidental exposures to *C. neoformans* in research settings, there is a paucity of literature available for guidance, with the exception of a historical case that led to the recommendation of prophylactic fluconazole treatment (200 mg/day for 14 days) (10). This recommendation is updated here to show that the prophylactic dose of fluconazole (200 mg/day) extended 4–8 weeks was curative for an active primary cutaneous *C. neoformans* infection acquired accidentally in the research laboratory.

In contrast to case 1, comparative genomic analysis of *C. neoformans* from an immunocompromised patient and their pet cockatoo’s feces provides evidence for consequential evolution in humans (21). Potential virulence-associated protein changes in *C. neoformans* were present in the patient and cockatoo samples or appeared in the patient and recurred upon mouse passage (21). Regarding case 1, it is conceivable that virulence features of the H99 lab strain (which is virulent in mice) had been acquired previously during human passage in the original Hodgkin’s lymphoma patient, and these features were retained during subsequent laboratory passage before transfer to the lab worker.

Cases 1–4 lacked known immunodeficiencies and were considered to be immunocompetent. However, lab workers with clinically confirmed cryptococcosis and unrecognized immunosuppressive risk factors, CNS disease, or fungemia may require more intense or extended antifungal therapy (22). However, traumatic inoculation cases with localized small lesions typically resolve with short courses of antifungal agents (up to 6–8 weeks) (7, 10). For case 1 reported here with an active local skin infection, 8 weeks of 200 mg/day fluconazole initiated on day 15 post-exposure and 7 days after onset of local symptoms was curative. Within the same 5-year time frame, three additional cases (cases 2–4) with assumed or potential exposures received prophylactic fluconazole within 24 h for 2–4 weeks (200 mg/day), and no symptoms developed. This report serves to encourage researchers and clinical workers in doubt of exposure to *C. neoformans* to seek further advice and consider prophylactic fluconazole, which is expected to inhibit the establishment of initial fungal infection and is justified in laboratory workers with potential exposures.

## Data Availability

Genome sequence data are deposited at SRA under PRJNA1370170.
